# Easily fabricated monolithic fluoropolymer chips for sensitive long-term absorbance measurement in droplet microfluidics†

**DOI:** 10.1039/d0ra05330a

**Published:** 2020-08-21

**Authors:** Adrian M. Nightingale, Sammer-ul Hassan, Kyriacos Makris, Wahida T. Bhuiyan, Terry J. Harvey, Xize Niu

**Affiliations:** Mechanical Engineering, Faculty of Engineering and Physical Sciences, University of Southampton Southampton SO17 1BJ UK a.nightingale@southampton.ac.uk; SouthWestSensor Ltd, Southampton Science Park, The Innovation Centre 2 Venture Road, Chilworth Southampton SO16 7NP UK

## Abstract

Maintaining a hydrophobic channel surface is critical to ensuring long-term stable flow in droplet microfluidics. Monolithic fluoropolymer chips ensure robust and reliable droplet flow as their native fluorous surfaces naturally preferentially wet fluorocarbon oils and do not deteriorate over time. Their fabrication, however, typically requires expensive heated hydraulic presses that make them inaccessible to many laboratories. Here we describe a method for micropatterning and bonding monolithic fluoropolymer flow cells from a commercially available melt-processable fluoropolymer, Dyneon THV 500GZ, that only requires a standard laboratory oven. Using this technique, we demonstrate the formation of complex microstructures, specifically the fabrication of sensitive absorbance flow cells for probing droplets in flow, featuring path lengths up to 10 mm. The native fluorous channel surface means the flow cells can be operated over extended periods, demonstrated by running droplets continuously through a chip for 16 weeks.

## Abstract 2

We present a widely accessible method for fabricating monolithic fluoropolymer microfluidic chips, which allows droplet absorbance measurement over multi-month periods.

## Graphical abstract

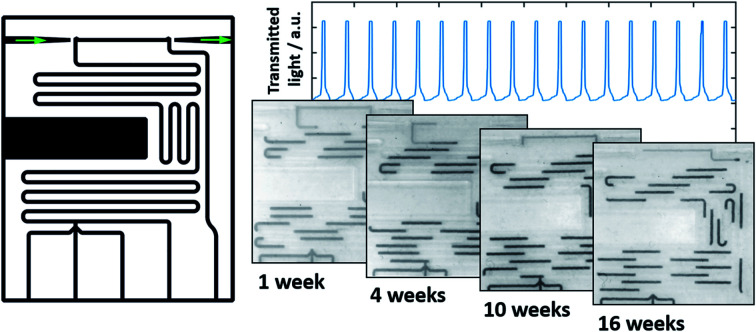

## Introduction

Droplet flow, in which a liquid phase travels as a succession of discrete self-contained droplets carried by a flow of (typically fluorous) oil, has the advantage of removing Taylor dispersion, enhancing mixing,^1^ preventing channel clogging,^2^ and can be used to isolate individual molecules or cells.^3^ A requirement for robust and resilient droplet flow is that channel walls must be consistently preferentially wetted by the continuous (oil) phase. If the droplet phase wets a section of wall the resulting pinning causes cross-contamination between droplets and a decrease in the uniformity of droplet size and composition.^4^ For the fluorous oils typically used as continuous phase in microfluidics, this necessitates fluorous channel surfaces. If non-fluorous materials are used for chip fabrication (*e.g.* poly(dimethyl siloxane), poly(methyl methacrylate), glass, cyclic olefin copolymer, *etc.*) additional surface treatments are required to render the channels fluorous. This adds an additional process step and yields a surface which can degrade if, for example, using elevated temperatures,^5^ or performing experiments over multiple days and weeks.^6,7^ Chips fabricated from fluoropolymers which feature native fluorous surfaces solve this problem, however fabrication of monolithic chips from common fluoropolymers (*e.g.* PTFE, FEP, PFA) is difficult due to poor suitability for direct machining, and high melting temperatures (*e.g.* 325 °C for PTFE, 315 °C for PFA), that mean expensive specialised equipment is required for moulding procedures.^8,9^ Consequently researchers often opt to assemble fluidic manifolds from off-the-shelf fluoropolymer capillary tubing^6,7,10^ as a more user-friendly option for long-term experimentation. In such systems fluidic architectures cannot be arbitrarily designed however, meaning complex structures such as the extended path length flow cells needed for sensitive absorbance measurement are unobtainable.

Optical absorption is one of the most extensively used analytical techniques as it is widely applicable, requires simple components, is label-free, is insensitive to matrix effects, and can draw on a large library of established assays. It is challenging to implement in microfluidics however, as the sensitivity of the measurement is directly proportional to the optical path length through the fluid. The simplest optofluidic geometries, where light is transmitted across the microfluidic channel, result in path lengths, and hence sensitivities, approximately two orders of magnitude lower than typical benchtop systems which have a standard path length of 10 mm. Consequently, several techniques have been developed to boost microfluidic path lengths^11^ such as cavity-enhanced optics,^11–13^ liquid waveguiding,^14^ and Z- or U-shaped flow cells.^15–17^ These techniques are routinely used in single-phase flow systems and extra considerations must be taken when applying them to droplet flow where lensing at the oil/aqueous interface can divert light away from the detector. Recently Yang *et al.*^18^ successfully measured droplet absorbance in flow and gained a 5.3-fold increase in absorbance path length relative to standard channel width, by squeezing droplets through narrow Z-shaped flow cells. By ensuring the flow cell volume was lower than that of the droplets they eliminated liquid–liquid interface lensing, with each droplet transition including a period where the droplet filled the flow cell and the main oil/aqueous interfaces were outside the light path. This system gave a significant increase in measurement sensitivity, however the use of a chip material which required surface modification means it is not ideally-suited to long-term droplet-based experimentation.^19^

Here we describe an easy method for fabricating monolithic fluoropolymer chips that offer increased optical path lengths and can be operated for months at a time. This was achieved using a commercially available melt-processable fluoropolymer, Dyneon THV 500GZ, and a new, simpler bonding method that obviates the need for expensive heated hydraulic presses previously reported for fabrication of fluoropolymer chips.^8,20–22^ The chips offer optical path lengths of up to 10 mm, the same as a standard benchtop spectrophotometer, and can be operated over multiple months without any degradation of channel surface and successfully maintaining consistent droplet flow. This opens up the possibility of long-term droplet-based experimentation that uses absorbance or indeed other techniques that require bespoke fluidic architectures.

## Experimental

### Chip fabrication

To form the native-fluorous absorption flow cells we microfabricated structures in Dyneon THV 500GZ (3M), a co-polymer made from tetrafluoroethylene, hexafluoropropylene, and vinylidene fluoride monomers. Like all fluoropolymers it is solvent resistant and hydrophobic (water contact angle 99 ± 2°)^20^ which is a requirement for stable oil/aqueous segmented flow. It is low cost (∼€50 per kg), optically transparent, and crucially its relatively low melting temperature for a fluoropolymer (165 °C) means it can be easily melt-processed using a standard laboratory oven with no need for the expensive hydraulic presses that feature in previous reports describing monolithic Teflon chips.^8,9,22^

**Fig. 1:**
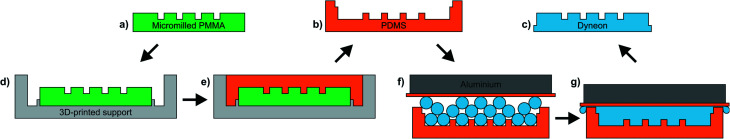
Cartoon illustrating how micromilled PMMA (a) is used to produce a PDMS mould (b) for melt-casting micropatterned Dyneon THV (c). The micromilled PMMA is placed in a 3D-printed support (d) and filled with liquid PDMS (e). The PDMS is cured and then removed to yield a mould (b). The PDMS mould is filled with Dyneon THV pellets, and a silicone sheet and aluminium weight placed on top (f). The Dyneon THV is melted (g) and, when cooled, removed to yield the final micropatterned substrate (c).

Microstructures were fabricated in Dyneon THV using a replica moulding strategy as illustrated in Fig. 1. The overall process involved using a microstructured poly(methyl methacrylate) (PMMA) master (Fig. 1a) to cast an intermediate poly(dimethylsiloxane) (PDMS) mould (Fig. 1b) which was then used to form the final Dyneon THV chip (Fig. 1c), in a method similar to that previously proposed by Begolo *et al.*^20^ The PMMA master was formed by milling microchannels into a 2 mm sheet of black PMMA (Sheet Plastics, Leicestershire, UK) using a LPKF Protomat S100 micromill (bits from ACS Industries Ltd, UK). Any standard microfluidic mould fabrication technique could be used (*e.g.* stereolithography, 3D printing) but here we chose micromilling as it produces high aspect ratio channels and vertical walls that, with smoothing, are highly suitable for optical applications.^23^ After milling, the mould was treated with chloroform vapour to re-melt the channel surfaces, remove all tool marks and yield an optically smooth surface (described in more detail in the ESI†).^23,24^ The smoothed PMMA master was then placed in a 3D-printed support (Fig. 1d, Veroclear, Connex Objet500), covered in PDMS (Fig. 1e, Dowsil Sylgard 184, elastomer : curing agent 20 : 1), degassed and cured (65 °C, >2 hours). The use of the 3D-printed support here ensured that the resulting PDMS mould had side walls to hold the Dyneon THV pellets during the next stage and determines the final thickness of the Dyneon THV substrate (3 mm). After curing, the PDMS mould was removed and filled with an excess of Dyneon THV pellets. To ensure the final chip was flat (important for later bonding), a ∼200 g aluminium block was placed on top, with a layer of 0.5 mm thick silicone (Silex Ltd, UK, 40° shore) placed between to allow easy removal after casting (Fig. 1f). This assembly was then left overnight in an oven at 190 °C (Thermo Scientific Heraeus) during which time the Dyneon THV melted and conformed to the PDMS mould (Fig. 1g). In contrast to previous reports describing microfabrication using Dyneon THV^20,21^ we found that a vacuum oven was not required. After cooling, the PDMS mould was peeled from the solid Dyneon THV chip (Fig. 1c) and then reused. PDMS moulds could typically be used 3–4 times before becoming brittle and tearing. For reproducible bonding in the next stage it is important that the compressive force, generated by the thermal expansion of the Dyneon THV and the tightening of the clamp bolts, is identical from chip-to-chip. Therefore, to ensure a uniform and reproducible compression from thermal expansion Dyneon THV substrates must be of reproducibly uniform thickness, hence the importance of using the aluminium weight during Dyneon THV casting (Fig. 1f and g). Additionally, as later described, the clamp bolts must be lightly tightened using a torque screwdriver so that the same compressive force can be applied each time.

**Fig. 2:**
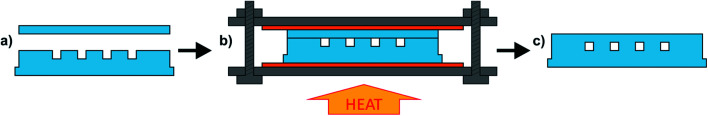
Cartoon illustrating how Dyneon THV substrates are bonded to yield monolithically sealed channels. Micropatterned and flat substrates are brought together (a), lightly clamped between two steel plates, with 0.5 mm silicone sheets (orange) as buffer layer, and heated to 160 °C (b), and left overnight during which time the two pieces fused (c).

The microchannels were sealed using a method previously described for bonding FEP or PFA^8^ (see Fig. 2). This method is cheaper, simpler, and more widely accessible than the previously reported Dyneon THV bonding methods^20,21^ which require the use of expensive heated hydraulic presses. In our method, the microstructured Dyneon THV substrate was brought together with an identically sized blank Dyneon THV sheet (Fig. 2a). The Dyneon THV parts were clamped between two steel plates using nuts and bolts, with 0.5 mm sheets of silicone (Silex Ltd, UK, 40° shore) placed between the Dyneon THV and steel (Fig. 2b) so that the chip could be easily removed at the end of the process. The nuts and bolts were lightly tightened to a torque of 0.2 Nm using a torque screwdriver (Q-Torq PRIME 150 FH) so that the Dyneon THV substrates were firmly and reproducibly contacted together but weren't appreciably compressed by the tightening of the bolts. The whole assembly was then placed in a 160 °C oven (just below the Dyneon THV's melting temperature of 165 °C) and left overnight. During heating the thermal expansion of the Dyneon THV provided the compression required to fuse the two pieces, while the finite expansion meant that the channels were not appreciably deformed, as is observed when using a constant pressure.^8^ When the assembly had been recovered from the oven, it was left to cool and then the finished chip extracted (Fig. 2c). Finally, to allow easy introduction and removal of fluid, PTFE tubing was inserted into recesses at each end of the main channel and fixed using epoxy glue (Araldite 2012).

**Fig. 3:**
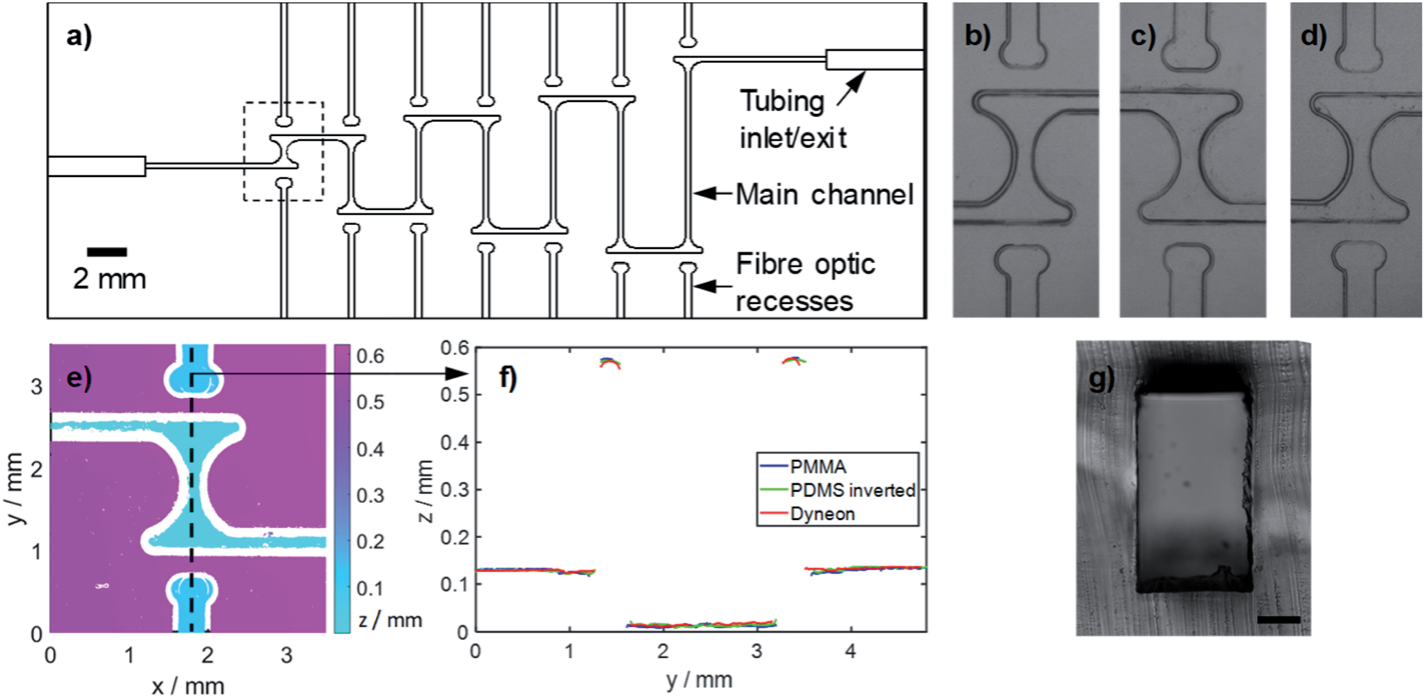
(a) Chip comprised of multiple flow cell structures with optical path lengths between 1.7 and 10 mm. (b–d) Microscope images of the shortest flow cell structure (dashed rectangle in (a)) fabricated in PMMA (b) and replica moulded in PDMS (c) and Dyneon THV (d). (e) Optical profilometry of the same structure in Dyneon THV. (f) Two-dimensional profiles along the fibre optic recesses and flow cell channel (dashed line shown in (f)) for each of the steps during fabrication. The PDMS profile is inverted for easier comparison with the PMMA master and finished Dyneon THV substrate. (g) Representative image of the main channel once bonded. The scale bar represents 100 μm.

To ascertain whether the Dyneon THV chips could be used to make long path length (multi-mm) flow cells for absorption measurement of droplets, we first created a Dyneon THV chip featuring an array of concatenated Z-shaped flow cells with increasing path lengths from 1.7 to 10 mm (Fig. 3a). In each case light was directed into, and collected from, the flow cell using fibre optics (320 μm outer diameter, 250 μm core, Thorlabs) which sat in milled recesses (340 μm square cross section). Fibre optics were inserted into each recess until they abutted the end, with the fibre optic fitting snugly within the recess. The alignment of the fibre optics and the fluidic channels are ensured as both the recesses and channels were simultaneously milled on the same master mould. The cross section of the main fluidic channel was 250 μm width by 500 μm height in each case, and the 90° turns within each “Z” were fabricated with a T-shape at each end to reduce droplet curvature at the point of light ingress/exit and hence loss of light due to lensing. Each turn of the Z featured exaggerated smoothed corners to ensure each droplet passed smoothly without breaking up. Away from the chip, the fibre optics were interfaced with LEDs (Cree CLM4B-GKW-CWBYA693, Farnell Onecall) and light-to-voltage converters (TSL257, Texas Advance Optical Solutions, Farnell Onecall) using bespoke 3D-printed couplers (printed in black poly(lactic acid) on an Ultimaker 3 fused deposition modelling printer, 3D CAD design files included in ESI†).

### Microstructure characterisation

Micropatterned structures were imaged using an Amscope SM-2T trinocular microscope with a Thorlabs DCC1240M USB camera. The *z*-dimensions of the micropatterned structures were measured by surface profilometry using a Proscan 2200 (Scantron Ltd) with a S12/1400 chromatic sensor (STIL). The sensor has a range of 1400 μm, working distance of 12.2 mm and spot size of 2.6 μm. Scans were performed with measurement steps of 5 μm in both the *x*- and *y*-dimensions.

Channel cross-sections of completed Dyneon THV chips were imaged by first cutting open a chip using a single edge razor blade (VWR) and hammer, and then slicing a sliver of the cross section using an fresh razor blade. The resulting cross section sliver was imaged using an Olympus IX81 inverted microscope and QImaging optiMOS camera, and dimensions quantified using pre-calibrated Olympus cellSens software.

### Chemicals

Unless otherwise stated, water was ultrapure grade (18.2 MΩ, Barnstead EASYpure RODI) and all chemicals were obtained from Sigma Aldrich, UK. A modified Griess assay for nitrite and nitrate measurement was formulated as previously described.^10,25^ Briefly, 1.25 g of vanadium(iii) chloride (99.0%, Alfa Aesar, UK) was added to a 250 ml volumetric flask along with 50 ml of ultrapure water to form a dark brown solution. 15 ml of concentrated (37%) hydrochloric acid was added, causing the solution to turn dark turquoise, then 1.25 g of sulfanilamide (≥99.0%) and 0.125 g of *N*-(1-naphthyl)ethylenediamine dihydrochloride (>98%) were added, dissolved, and the solution finally made up to the volumetric mark using ultrapure water. A 100 mM stock solution of sodium nitrite was made by dissolving 1.725 g of sodium nitrite (≥99.0%) in ultrapure water in a 250 ml volumetric flask and then diluted as required to make up lower concentrations. Fluorinert FC40 oil was obtained from Acota Ltd, UK.

### Fluid handling

All fluids were pumped using peristaltic pumps built in-house.^25,26^ For initial flow cell testing, droplets were generated in a separate PDMS-based T-junction chip and then flowed into the Dyneon THV chip. The droplet generation chip was fabricated using standard replica moulding methods, as previously reported.^25^ Droplets were generated using the anti-phase peristaltic pumping method,^26^ whereby the oil and aqueous phases are delivered in time-separated alternate pulses, meaning droplet size, composition, and generation frequency is mechanically determined by the design of the pump and is independent of oil/aqueous interfacial tension.

## Results

Replica moulding relies on all features being faithfully reproduced in each individual moulding stage. To assess the fidelity of replication, a specific flow cell (highlighted by dashed box in Fig. 3a) was measured in each substrate (PMMA, PDMS, Dyneon THV) in the *x*–*y* plane using bright-field microscopy (Fig. 3b–d) and in the *z*-direction using optical profilometry (Fig. 3e and f). The *x*–*y* feature definition for each mould (Fig. 3b and c) and final Dyneon THV substrate (Fig. 3d) match closely, with the only visual difference being the inversion of the PDMS mould (Fig. 3c). Fig. 3e shows the feature heights for the flow cell as measured in the Dyneon THV substrate, with the corresponding PMMA and PDMS measurements shown in ESI Fig S1.† A 2-dimensional profile along the central axis of the flow cell (dashed line in Fig. 3e) encompassing the main channel, fibre optic recess and substrate surface, is shown in Fig. 3f along with the equivalent profiles for the PMMA and PDMS moulds, with the PDMS profile inverted for easier comparison. The profiles match well indicating the topology of the PMMA master was faithfully replicated in both the PDMS mould and the final Dyneon THV substrate. After bonding the features showed minimal deformation as shown, for example, by the cross section of the main fluidic channel in Fig. 3g. The potential of the replica moulding technique to replicate much finer features is apparent when imaging substrates cast from an unsmoothed PMMA master, where all the tooling marks (normally removed by solvent smoothing) are visible in the finished Dyneon THV substrate (see ESI Fig. S2†).

The ability of the flow cells to deliver accurate absorbance measurements was assessed by generating coloured droplets off-chip, and then flowing them through the chip. The colour of the droplets was generated by implementing a colourimetric assay for nitrite. Each droplet contained an equal volume of nitrite standard and Griess reagent, which reacted to form a purple coloured diazonium product with an absorbance maximum at ∼525 nm. The droplets were generated using our previously-reported anti-phase peristaltic pumping approach,^26^ whereby oil and aqueous phases are supplied in time-separated pulses. In this way droplet size is determined solely by the volume supplied in each aqueous pulse and allowed us to easily ensure that droplets could be generated of pre-determined sizes (1.6 μl) that could fill every flow cell.

**Fig. 4:**
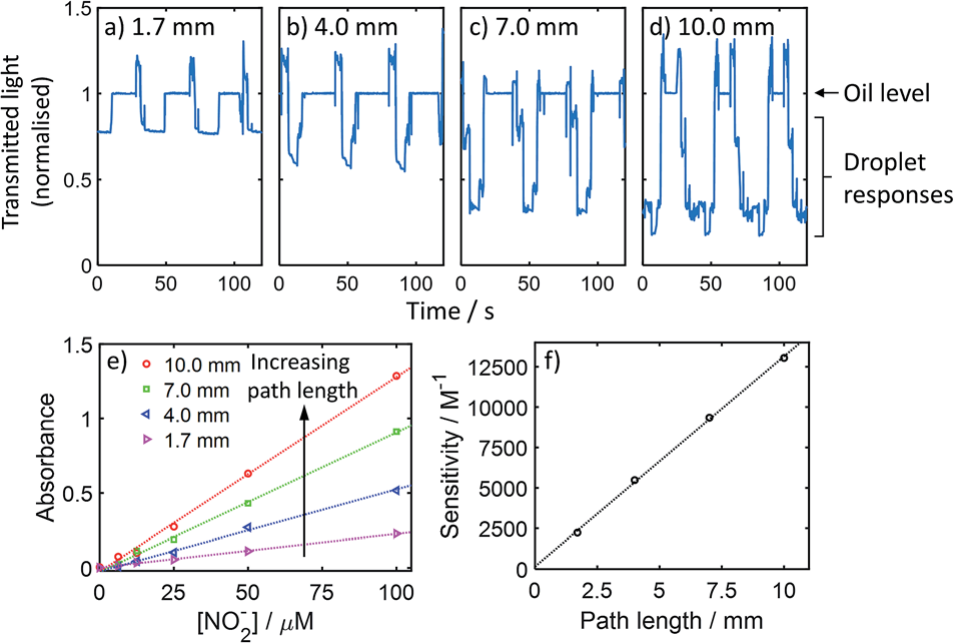
Optical measurement of droplets passing through the chip shown in Fig. 3(a). Transmitted light signal measured as three coloured droplets and accompanying oil segments pass through the 1.7 mm (a), 4.0 mm (b), 7.0 mm (c), and 10.0 mm (d) path length flow cells. In each case the data is normalised to the light level measured through the oil segments. (e) Calibration curves for the Griess colorimetric nitrite assay, for optical path lengths of 1.7 mm, 4.0 mm, 7.0 mm and 10.0 mm. (f) Sensitivity of each calibration shown in (e) shown as a function of path length.

The droplets passed through the chip without breakup and filled each channel (representative image shown in ESI Fig. S3†). This is important as an accurate absorbance measurement requires the droplet to completely fill the flow cell at some point during its transition, thus removing potential interference from lensing at the oil/droplet interface. As the droplet flow passed through each flow cell the transmitted light exhibited a characteristic waveform (Fig. 4a–d) with high light transmission when oil filled the flow cells, and lower levels when the coloured droplets filled the flow cell and absorbed a proportion of the light. Increasing path length from 1.7 to 10 mm (Fig. 4a–d) caused greater decreases in light levels in keeping with the Beer–Lambert law:*A* = *εcl*where *A* is the absorbance, *ε* is the molar extinction coefficient, *c* is the concentration of the absorbing species, and *l* is the optical path length. The shape of the waveform also changed – from having long plateaus (periods where a single fluid phase exclusively occupies the flow cell) and sharp transitions between these plateaus (Fig. 4a) to shorter plateaus and structured transitions (Fig. 4d). This is consistent with the increasing flow cell volume causing shorter periods during which each segment (oil or aqueous) can fill the whole flow cell, and longer periods when the oil/aqueous interface travels through the flow cell. The structured, stepwise nature of the transitions in longer flow cells is consistent with the pulsed peristaltic pumping moving the droplets through in discrete steps, and can be correlated to pump rotation speed and pulse timing (data not shown).

To demonstrate the increased measurement sensitivity with increasing path length in more detail, we ran different concentrations of nitrite through the system and obtained separate calibration curves for flow cells with path lengths ranging from 1.7 to 10 mm (Fig. 4e). In each case a linear response was obtained, with the gradient (corresponding to measurement sensitivity) increasing with path length. The sensitivity (gradient) is defined by the Beer–Lambert law as *εl* and hence should vary linearly with path length. This was found to be the case (see Fig. 4f) and the gradient, corresponding to *ε*, was 13 060 M^−1^ cm^−1^ which is consistent with our previous testing using the same assay in short path length flow cells.^10^ The increased path lengths demonstrated here increase sensitivity in colourimetric assays and, by maximising signal : noise ratio, lead to increased precision and lower limits of detection.^27^ Longer path lengths will also reduce the measurement ceiling, but shorter flow cells can be used in combination with longer flow cells^28,29^ to maximise the measurable range.

**Fig. 5:**
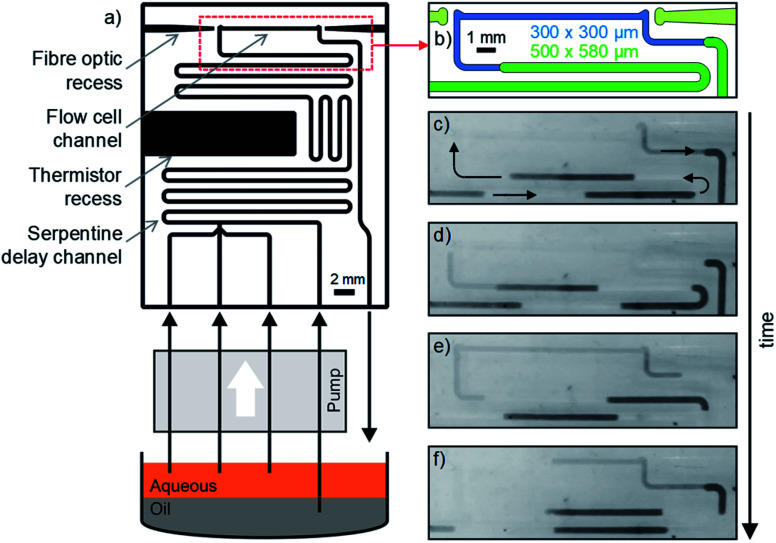
(a) Cartoon showing the chip design and experimental setup for long-term testing of the resilience of the chip surface. The chip features a 10 mm long path length with a reduced channel cross-section to extend the length of each droplet, shown in (b). Representative images showing a droplet being elongated as it passes through the flow cell without any breakup are shown in (c–f).

The use of a fluoropolymer as the chip material means that droplets can, in principle, flow through the chip indefinitely without fear of droplets pinning to the walls, and causing subsequent cross-contamination and disordered flow.^4^ To test this, we fabricated a chip where droplets could be continuously and indefinitely generated and flowed through a 10 mm absorbance cell. The chip (Fig. 5a) was designed to be representative of a device that might be used for colourimetric assays in droplet flow, hence it not only included an optical flow cell but also a T-junction for droplet generation, serpentine delay channels, and a recess for a thermistor to monitor chip temperature (Fig. 5a). To increase the residence time in the serpentine delay section, whilst also ensuring the droplets filled the flow cell, the cross section of the fluidic channel was larger for the serpentine section than the flow cell: (500 × 580 μm *vs.* 300 × 300 μm width × height, see Fig. 5b). This meant that as the droplets moved into the flow cell they approximately tripled in length to ensure that the flow cell was completely filled (as shown in Fig. 5c–f, and ESI video SV1†). As before, the chip was supplied with fluid using a peristaltic pump generating droplets every 9 s with volumes of 1.5 μl. Each of the three aqueous inlets was supplied with dyed water to generate droplets that were clearly discernible, and so that any pinned aqueous fluid could be visibly identified. On exiting the chip, the droplet flow was collected in a sample tube where the fluids gravimetrically separated before being recycled (as illustrated in Fig. 5a), ensuring the system could be run indefinitely without manual intervention.

**Fig. 6:**
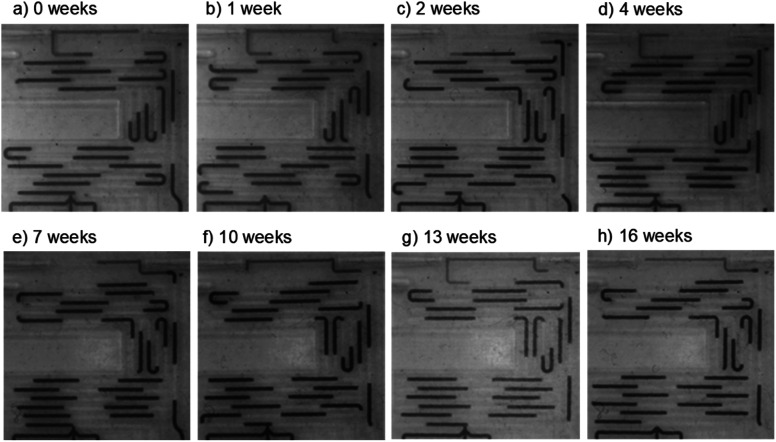
Images showing the chip at different points during long-term testing, from *t* = 0 (a) to *t* = 16 weeks (h). In each case the droplets are uniformly sized and there is no evidence of droplet pinning or breakup.

The setup was left running with a computer-controlled microscope periodically taking images (every 15 minutes) and videos of the chip (initially every day, then every week). The system ran continuously for over 16 weeks, at which point the experiment was ended due to laboratory shutdown accompanying the 2020 global coronavirus pandemic. Over time the droplet size decreased by approximately 20% due to compression of the peristaltic pump tubing and hence a reduction in the volume of fluid delivered in each pulse (see *e.g.*Fig. 6a*vs.*Fig. 6h), but crucially the droplets continued to flow without wetting the chip wall. This is shown in the representative images taken after 0, 1, 2, 4, 7, 10, 13 and 16 weeks (Fig. 6) and in videos of the flow taken after 1, 2, 4, 6, 8, 10, 13 and 16 weeks (ESI video SV2†) where the droplets can be seen to be uniformly sized with no evidence of residual aqueous fluid on the channel walls. The absorbance flow cell was still performing well after the 16 week period, producing a uniform reproducible waveform signal (ESI, Fig. S4†) consistent with droplets of identical size passing through cleanly and filling the light path during each transition. This shows that the chip can be confidently used over multi-month periods without droplet pinning or breakup.

## Conclusions

Here we have shown a simple method to fabricate 10 mm path length absorbance flow cells, the standard path length used in benchtop spectrophotometers, in monolithic fluoropolymer chips. These allow droplets to be measured with high sensitively and can be used over multi-month periods. It should be noted that these structures have not been extensively optimised, so while the droplets used here are relatively large (>1 μl) it will be possible to target smaller volumes by reducing cross section area of the flow cells' fluidic channel.

The sensitive and robust measurement of droplet absorbance has direct implications on droplet microfluidic systems that use or could use absorbance measurement in long-term experimentation, with the increase in sensitivity allowing lower detection limits and increased precision. This, in turn, increases the range of potential applications and analytes for absorbance-based droplet microfluidic systems.^7,10^ More generally, however, this shows how arbitrary microfluidic architectures can be easily implemented in monolithic fluoropolymer chips without need for expensive laboratory equipment, and opens up for a range of different analytical methods and functionalities to be robustly addressed with droplet-based methods.

## Conflicts of interest

There are no conflicts to declare.

## Supplementary Material

RA-010-D0RA05330A-s001

RA-010-D0RA05330A-s002

RA-010-D0RA05330A-s003

RA-010-D0RA05330A-s004

RA-010-D0RA05330A-s005

## References

[cit1] Song H., Chen D. L., Ismagilov R. F. (2006). Reactions in Droplets in Microfluidic Channels. Angew. Chem., Int. Ed..

[cit2] Nightingale A. M., Krishnadasan S. H., Berhanu D., Niu X., Drury C., McIntyre R., Valsami-Jones E., deMello J. C. (2011). A stable droplet reactor for high temperature nanocrystal synthesis. Lab Chip.

[cit3] Brouzes E., Medkova M., Savenelli N., Marran D., Twardowski M., Hutchison J. B., Rothberg J. M., Link D. R., Perrimon N., Samuels M. L. (2009). Droplet microfluidic technology for single-cell high-throughput screening. Proc. Natl. Acad. Sci. U. S. A..

[cit4] Debon A. P., Wootton R. C. R., Elvira K. S. (2015). Droplet confinement and leakage: causes, underlying effects, and amelioration strategies. Biomicrofluidics.

[cit5] Chan E. M., Alivisatos A. P., Mathies R. A. (2005). High-Temperature Microfluidic Synthesis of CdSe Nanocrystals in Nanoliter Droplets. J. Am. Chem. Soc..

[cit6] Cybulski O., Jakiela S., Garstecki P. (2016). Whole Teflon valves for handling droplets. Lab Chip.

[cit7] Horka M., Sun S. W., Ruszczak A., Garstecki P., Mayr T. (2016). Lifetime of Phosphorescence from Nanoparticles Yields Accurate Measurement of Concentration of Oxygen in Microdroplets, Allowing One To Monitor the Metabolism of Bacteria. Anal. Chem..

[cit8] Ren K. N., Dai W., Zhou J. H., Su J., Wu H. K. (2011). Whole-Teflon microfluidic chips. Proc. Natl. Acad. Sci. U. S. A..

[cit9] Zheng H., Wang W. Z., Li X. J., Wang Z. H., Hood L., Lausted C., Hu Z. Y. (2013). An automated Teflon microfluidic peptide synthesizer. Lab Chip.

[cit10] Nightingale A. M., Hassan S.-u., Warren B. M., Makris K., Evans G. W. H., Papadopoulou E., Coleman S., Niu X. (2019). A Droplet Microfluidic-Based Sensor for Simultaneous *in situ* Monitoring of Nitrate and Nitrite in Natural Waters. Environ. Sci. Technol..

[cit11] Rushworth C. M., Davies J., Cabral J. T., Dolan P. R., Smith J. M., Vallance C. (2012). Cavity-enhanced optical methods for online microfluidic analysis. Chem. Phys. Lett..

[cit12] Rushworth C. M., Jones G., Fischlechner M., Walton E., Morgan H. (2015). On-chip cavity-enhanced absorption spectroscopy using a white light-emitting diode and polymer mirrors. Lab Chip.

[cit13] Zhu J. M., Shi Y., Zhu X. Q., Yang Y., Jiang F. H., Sun C. J., Zhao W. H., Han X. T. (2017). Optofluidic marine phosphate detection with enhanced absorption using a Fabry–Pérot resonator. Lab Chip.

[cit14] Waterbury R. D., Yao W., Byrne R. H. (1997). Long pathlength absorbance spectroscopy: trace analysis of Fe(ii) using a 4.5 m liquid core waveguide. Anal. Chim. Acta.

[cit15] Petersen N. J., Mogensen K. B., Kutter J. P. (2002). Performance of an in-plane detection cell with integrated waveguides for UV/vis absorbance measurements on microfluidic separation devices. Electrophoresis.

[cit16] Sieben V. J., Floquet C. F. A., Ogilvie I. R. G., Mowlem M. C., Morgan H. (2010). Microfluidic colourimetric chemical analysis system: application to nitrite detection. Anal. Methods.

[cit17] Luy E. A., Morgan S. C., Creelman J. J., Murphy B. J., Sieben V. J. (2020). Inlaid microfluidic optics: absorbance cells in clear devices applied to nitrite and phosphate detection. J. Micromech. Microeng..

[cit18] Yang T. J., Stavrakis S., deMello A. (2017). A High-Sensitivity, Integrated Absorbance and Fluorescence Detection Scheme for Probing Picoliter-Volume Droplets in Segmented Flows. Anal. Chem..

[cit19] Kaminski T. S., Scheler O., Garstecki P. (2016). Droplet microfluidics for microbiology: techniques, applications and challenges. Lab Chip.

[cit20] Begolo S., Colas G., Viovy J. L., Malaquin L. (2011). New family of fluorinated polymer chips for droplet and organic solvent microfluidics. Lab Chip.

[cit21] Aboud N., Ferraro D., Taverna M., Descroix S., Smadja C., Tran N. T. (2016). Dyneon THV, a fluorinated thermoplastic as a novel material for microchip capillary electrophoresis. Analyst.

[cit22] Shen B., Wu H. (2016). Aqueous and Nonaqueous Electrochemical Sensing on Whole-Teflon Chip. ACS Sens..

[cit23] Ogilvie I. R. G., Sieben V. J., Floquet C. F. A., Zmijan R., Mowlem M. C., Morgan H. (2010). Reduction of surface roughness for optical quality microfluidic devices in PMMA and COC. J. Micromech. Microeng..

[cit24] Hassan S.-u., Morgan H., Zhang X., Niu X. (2015). Droplet Interfaced Parallel and Quantitative Microfluidic-Based Separations. Anal. Chem..

[cit25] Nightingale A. M., Hassan S.-u., Evans G. W. H., Coleman S. M., Niu X. (2018). Nitrate measurement in droplet flow: gas-mediated crosstalk and correction. Lab Chip.

[cit26] Nightingale A. M., Evans G. W. H., Xu P., Kim B. J., Hassan S.-u., Niu X. (2017). Phased peristaltic micropumping for continuous sampling and hardcoded droplet generation. Lab Chip.

[cit27] Loock H.-P., Wentzell P. D. (2012). Detection limits of chemical sensors: applications and misapplications. Sens. Actuators, B.

[cit28] Beaton A. D., Cardwell C. L., Thomas R. S., Sieben V. J., Legiret F.-E., Waugh E. M., Statham P. J., Mowlem M. C., Morgan H. (2012). Lab-on-Chip Measurement of Nitrate and Nitrite for *In Situ* Analysis of Natural Waters. Environ. Sci. Technol..

[cit29] Clinton-Bailey G. S., Grand M. M., Beaton A. D., Nightingale A. M., Owsianka D. R., Slavik G. J., Connelly D. P., Cardwell C. L., Mowlem M. C. (2017). A Lab-on-Chip Analyzer for *in situ* Measurement of Soluble Reactive Phosphate: Improved Phosphate Blue Assay and Application to Fluvial Monitoring. Environ. Sci. Technol..

