# Supplementary material for: Easily fabricated monolithic fluoropolymer chips for sensitive long-term absorbance measurement in droplet microfluidics
Source: RSC Adv. 2020 Aug 21;10(51):30975–81. doi: 10.1039/d0ra05330a (PMC9056331; doi:10.1039/d0ra05330a)
Supplement: RA-010-D0RA05330A-s001 [file RA-010-D0RA05330A-s001.pdf]

Electronic Supplementary Material (ESI) for RSC Advances.
This journal is © The Royal Society of Chemistry 2020

Easily fabricated monolithic fluoropolymer chips for sensitive long-term absorbance measurement in droplet microfluidics

Adrian M. Nightingale,[a] Sammer-ul Hassan,[a] Kyriacos Makris,[b] Wahida T. Bhuiyan,[a] Terry J. Harvey,[a] and Xize Niu[a,b]*

[a]Mechanical Engineering, Faculty of Engineering and Physical Sciences, University of Southampton, Southampton, SO17 1BJ, United Kingdom.

[b]SouthWestSensor Ltd, Southampton Science Park, The Innovation Centre, 2 Venture Road, Chilworth, Southampton, SO16 7NP, United Kingdom.

Solvent smoothing of PMMA moulds

To remove the tool marks from the micromilled PMMA master a solvent vapour smoothing method was used. An approximately 1 cm depth of chloroform was placed in a petridish along with four steel bolts, which acted as supports for the PMMA master. The master was placed face-down on the bolts (one at each corner), the lid placed back on the dish, and then left for three minutes. The master was then taken from the dish and left face-up for all absorbed vapour to evaporate. After treatment the tooling marks within the milled channels had been visibly smoothed, as shown in Fig. S5.

Supplementary figures

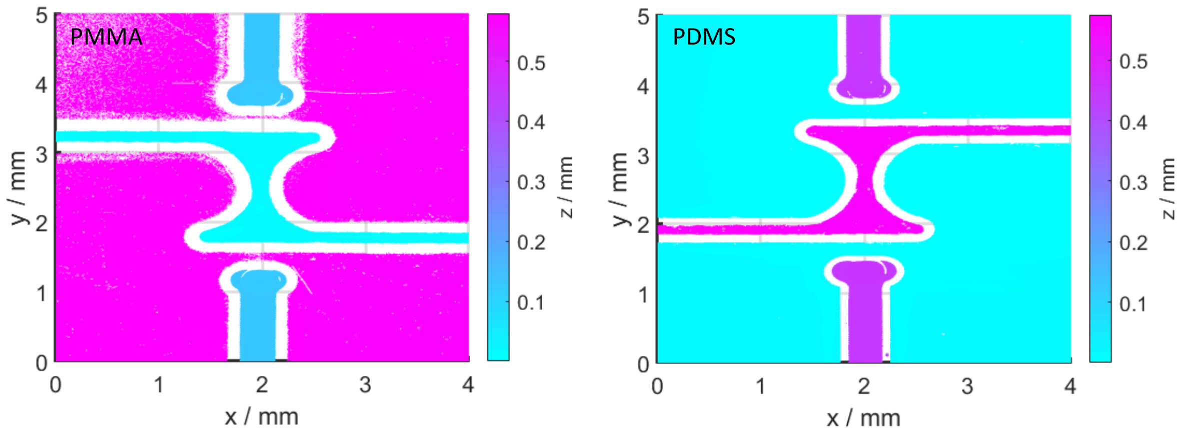

Figure S1: Optical profilometry results for the PMMA master and PDMS moulds. In each case the results correspond to the same structure shown in Fig. 3 in the main manuscript.

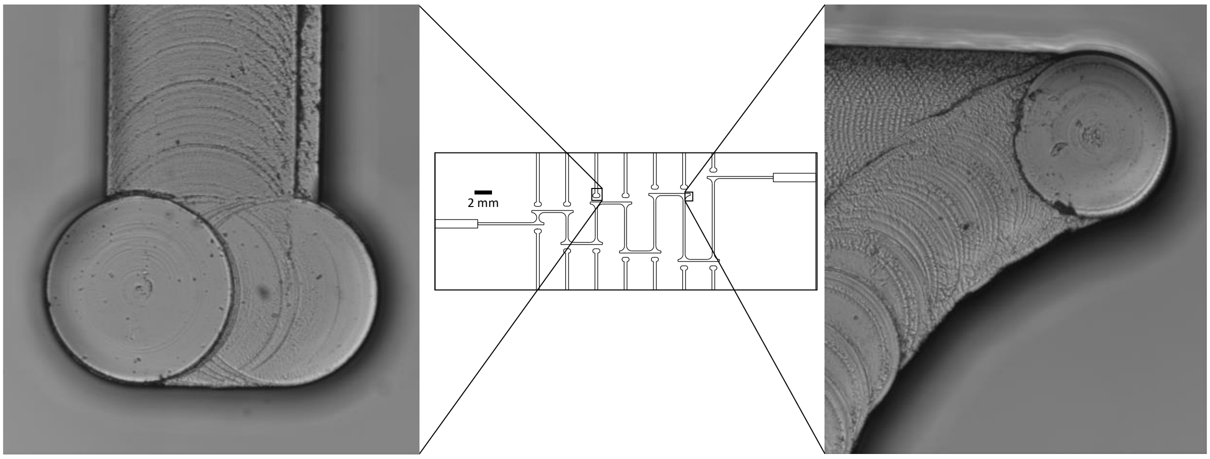

Figure S2: Images of microstructures from a Dyneon chip cast from an unsmoothed PMMA master mould. All tooling marks from the micromilling of the structures have been replicated from the master.

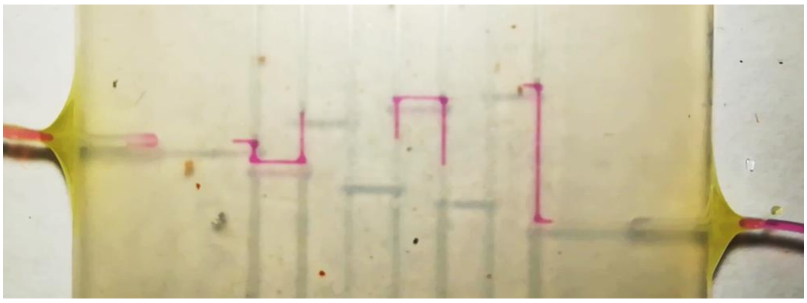

Figure S3: Image of droplets passing through the concatenated flow cell chip (shown schematically in Fig. 3 in the main manuscript). Droplets flowed through without breakup. Here a droplet can be seen filling the entire volume of the largest (10 mm path length) flow cell.

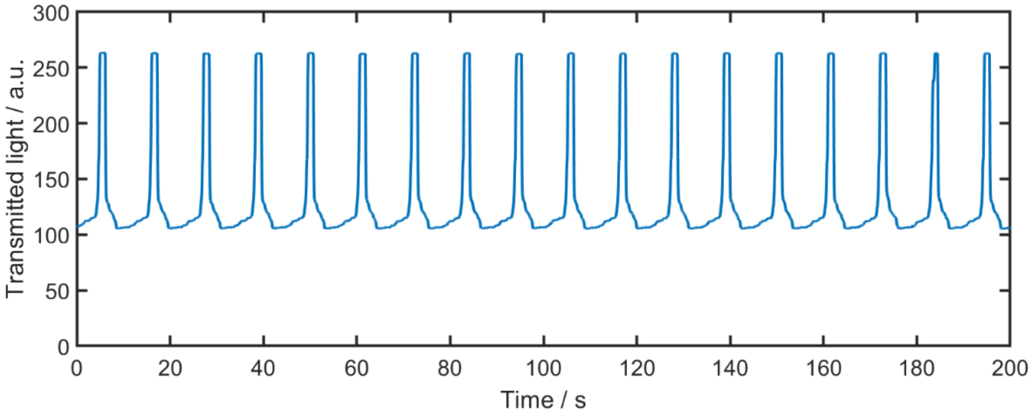

Figure S4: Transmitted light through the flow cell of the long-term test chip (shown in Fig. 5) after 16 weeks of operation. The regular wave-like signal indicates the reproducible droplet and oil segment size, with the plateau for each segment indicating that each segment fills the flow cell.

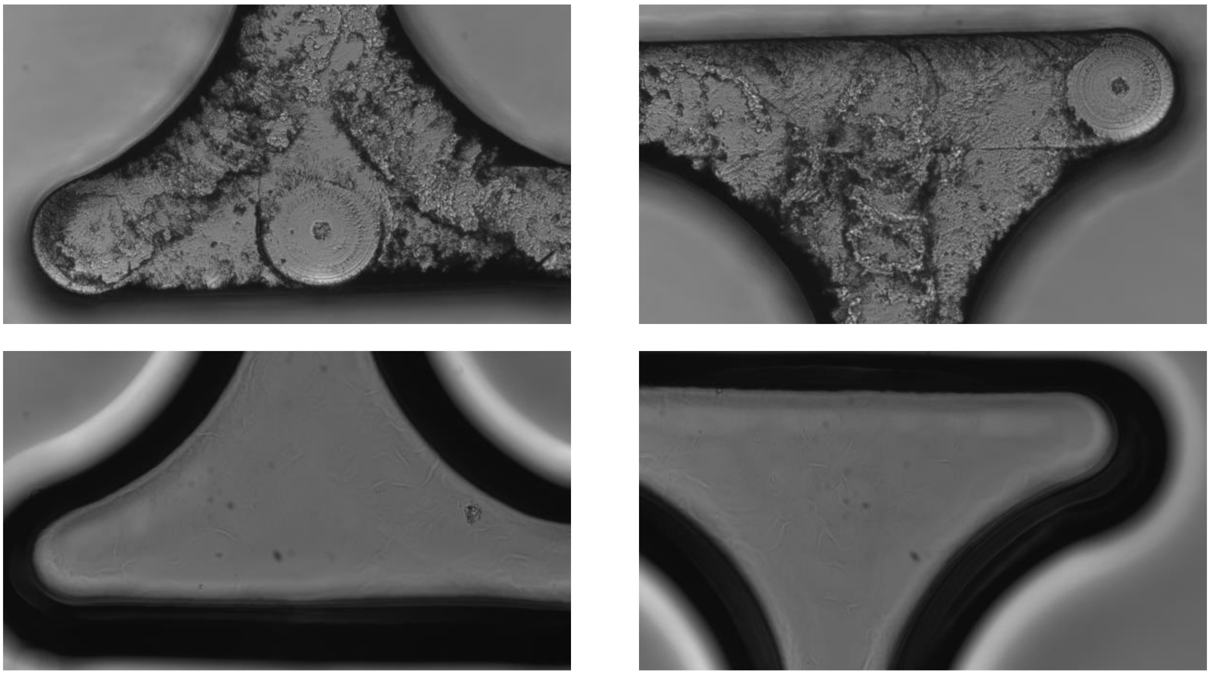

Figure S5: PMMA microstructures before (top) and after (bottom) solvent smoothing.
